# A144 EOSINOPHILIC GASTRITIS: A SERIES OF CHILDREN FROM BC CHILDREN’S HOSPITAL USING NASPGHAN/ESPGHAN CRITERIA

**DOI:** 10.1093/jcag/gwae059.144

**Published:** 2025-02-10

**Authors:** A Greaves, J Bush, V Avinashi

**Affiliations:** Pediatric Gastroenterology, BC Children’s Hospital, Vancouver, BC, Canada; Pediatric Gastroenterology, BC Children’s Hospital, Vancouver, BC, Canada; BC Children’s Hospital, Vancouver, BC, Canada

## Abstract

**Background:**

Eosinophilic gastritis (EoG) involves eosinophilic infiltration of the stomach, leading to symptoms without identifiable secondary causes. It can occur with Eosinophilic Esophagitis (EoE) or independently (non-EoE EGID). Common symptoms include abdominal pain, vomiting, fatigue, and edema. Recent North American Society of Pediatric Gastroenterology, Hepatology and Nutrition/European Society of Pediatric Gastroenterology, Hepatology and Nutrition (NASPGHAN/ESPGHAN) criteria (Jan 2024) define EoG as >30 eosinophils per high-power field (hpf).

**Aims:**

This study aimed to identify and review cases of gastric eosinophilia at BC Children’s Hospital over a five-year period, examining clinical details of those meeting the new NASPGHAN/ESPGHAN criteria.

**Methods:**

Pathology reports from August 2019-2024 were reviewed to identify cases meeting EoG criteria. Clinical details, including symptoms, EoE association, food allergies, laboratory findings, and treatments, were analyzed.

**Results:**

A total of 31 cases were identified, with 4 having secondary causes of eosinophilia (ulcers, liver transplant, Crohn’s disease). Of the remaining 27 EGID cases, 8 had both EoE and EoG, while 18 had non-EoE EGID (13 confined to the stomach, 5 involving both the stomach and duodenum).

1. Mean age: 6.3 years (EoE-EGID) vs. 8.7 years (non-EoE EGID)

2. Gender: EoE-EGID showed male predominance (67%), while non-EoE EGID was equally prevalent in males and females (50%).

3. IgE-based food allergies were common in both groups (58% vs. 67%).

4. Iron deficiency anemia was significantly more prevalent in non-EoE EGID (61% vs. 11%) and hypoalbuminemia (39% vs. 11%).

5. Nausea and vomiting were more frequent in EoE (55%), with dysphagia at 44%. Non-EoE cases had these symptoms less frequently (22%).

6. Treatment varied, including steroids, proton pump inhibitors (PPI), and dietary elimination. 80% of EoE-EGID cases were prescribed steroids or diet, compared to 35% and 45% for non-EoE EGID.

**Conclusions:**

Eosinophilic gastritis was found in 27 patients in the past 5 years. Symptoms are quite non-specific and even overlap between EoE and non-EoE EGID cases. While EoG patients are more likely to present with iron deficiency anemia and hypoalbuminemia, these are far from universal. Further research is necessary to better understand the natural history of this condition which can also better guide disease specific treatment.

**Funding Agencies:**

None

